# Inspiratory muscle training as an adjunct to standard cardiac rehabilitation in patients with LVEF < 50%: a randomized controlled trial

**DOI:** 10.1186/s12872-026-05889-4

**Published:** 2026-04-21

**Authors:** Jin-hong Xu, Li-xian Zheng, Ni-bing Zheng, Xing-xing Hu, Bin Chen

**Affiliations:** Department of Cardiology, Cardiac Rehabilitation Center, Shengli Clinical Medical College of Fujian Medical University, Fuzhou University Affiliated Provincial Hospital, Fujian Provincial Hospital, No. 134 Dongjie Street, Fuzhou, 350001 China

**Keywords:** Heart failure, Cardiac rehabilitation, Inspiratory muscle training, Exercise tolerance, Quality of life

## Abstract

**Background:**

Impaired inspiratory muscle strength is prevalent in heart failure (HF). Despite standard aerobic and resistance training, patients often experience residual exercise intolerance. Adding inspiratory muscle training (IMT) appears promising, but its aprecise additive value remains unclear, particularly in contemporary cohorts with LVEF < 50%.

**Methods:**

We randomized 65 HF patients (LVEF < 50%) to standard aerobic and resistance training (AR group, *n* = 32; 60 min/session) or AR plus IMT (ARIS strategy, *n* = 33; 90 min/session). Both protocols prescribed 36 sessions (3 sessions/week for 12 weeks). The primary outcome was the change in the Kansas City Cardiomyopathy Questionnaire (KCCQ) Overall Summary Score. Data were analyzed according to the intention-to-treat (ITT) principle.

**Results:**

Full completion of all 36 prescribed sessions was achieved by 53.1% (AR) and 57.6% (ARIS) of patients, yielding an actual time-adjusted training density of 2.38 and 2.53 sessions/week, respectively. In the primary outcome analysis, the ARIS strategy demonstrated greater KCCQ Overall Summary Score improvement (between-group difference: +6.3 points; *P* = 0.006), exceeding the minimal clinically important difference (MCID) of 5 points. The ARIS strategy also yielded significantly greater increases in 6-minute walk distance (+ 33.9 m; *P* = 0.016) and maximal inspiratory pressure (+ 2.0 kPa; *P* < 0.001). Regarding cardiac structure, no significant between-group differences were observed for changes in LVEF (*P* = 0.818) or left ventricular end-diastolic volume (LVEDV) (*P* = 0.990).

**Conclusions:**

In HF patients (LVEF < 50%), the extended multimodality ARIS strategy provides clinically significant improvements in quality of life and functional capacity compared to standard training. These incremental benefits likely stem from the combined addition of IMT and higher exercise volume, occurring without significant between-group differences in macro-structural cardiac remodeling. Adequately powered studies are needed to confirm efficacy in the HFmrEF subgroup.

**Trial registration:**

Chinese Clinical Trial Registry ChiCTR2500108124. Retrospectively registered on 25 August 2025.

**Supplementary Information:**

The online version contains supplementary material available at 10.1186/s12872-026-05889-4.

## Introduction

Heart failure (HF) represents an escalating global public health challenge, affecting more than 64 million individuals worldwide and imposing substantial morbidity and mortality burdens [1, 2]. Regardless of left ventricular ejection fraction (LVEF), patients with HF consistently experience progressive exercise intolerance, dyspnea, and impaired quality of life [3, 4]. Exercise-based cardiac rehabilitation is recognized as a cornerstone of comprehensive HF management and receives a Class IA recommendation from major international guidelines [5, 6]. The traditional “gold standard” for rehabilitation predominantly consists of aerobic exercise combined with resistance training (AR), aimed at improving functional capacity and skeletal muscle conditioning [7, 8].

Despite its established role, the clinical benefits of standard AR remain suboptimal for many patients. The landmark HF-ACTION trial demonstrated the safety of aerobic training; however, modest significant reductions in both the primary composite endpoint (all-cause mortality or hospitalization) and the secondary endpoint (cardiovascular mortality or HF hospitalization) were only observed after adjustment for highly prognostic predictors [9]. Consequently, despite receiving standard aerobic and resistance training, patients often experience residual exercise intolerance. This limitation may reflect the shortcomings of traditional two-dimensional exercise prescriptions (aerobic plus resistance), which overlook a critical pathophysiological target: the respiratory muscles. Approximately 30% to 50% of patients with HF exhibit impaired inspiratory muscle strength [10, 11], which triggers a metaboreflex that sympathetically restricts blood flow to locomotor muscles, thereby exacerbating fatigue and exercise intolerance [12–14]. Addressing this deficit may therefore be essential to achieving further improvements in clinical outcomes.

Emerging evidence suggests that multidimensional rehabilitation strategies outperform traditional single- or dual-mode protocols. The REHAB-HF trial demonstrated that a comprehensive intervention incorporating strength, balance, and functional training significantly improved physical function in older patients with HF [15]. Building on this multidomain concept, the ARISTOS-HF trial proposed combining inspiratory muscle training (IMT) with aerobic and resistance exercises, suggesting potential superiority over standard care [16]. However, the definitive role of IMT remains limited by two critical shortcomings in prior research. First, most studies, including ARISTOS-HF, used time-matched designs that reduce the duration of standard components to accommodate IMT [16]. Although scientifically rigorous for isolating variables, this approach potentially dilutes the established benefits of standard training. Second, enrollment is typically restricted to patients with HFrEF, limiting generalizability to the broader population with reduced ejection fraction, including those with mildly reduced ejection fraction (HFmrEF) [6, 17].

To address these gaps, we designed a randomized controlled trial using a time-additive strategy, in which IMT was superimposed on a full-duration standard AR protocol. This design ensured that the background standard of care remained uncompromised and allowed assessment of IMT as a true adjunct therapy. We also expanded the inclusion criteria to patients with LVEF < 50%, thereby broadening the phenotypic spectrum of the cohort. We hypothesized that, in this expanded population, the addition of IMT to standard rehabilitation would result in clinically meaningful improvements in quality of life and functional capacity. Additionally, as an exploratory objective, we investigated whether this strategy would induce favorable changes in cardiac structure compared with standard training alone.

## Methods

### Study design and oversight

We conducted a prospective, single-center, randomized controlled trial at the Cardiac Rehabilitation Center of Fuzhou University Affiliated Provincial Hospital between January and September 2025. This trial was designed a priori as a nested, randomized sub-study operating within the broader institutional framework of the overarching clinical project, ‘Real-world study of lifestyle intervention based on digital therapeutics for cardiometabolic diseases.’

While the parent registry (ChiCTR2500108124) encompasses a broader observational cohort, our specific sub-study was pre-planned to employ a strictly prospective randomized controlled methodology. This allowed us to rigorously evaluate a targeted combined intervention (ARIS strategy) in a vulnerable subset of patients with HF. Ethical oversight was provided under the umbrella protocol approved by the hospital’s Ethics Committee (Approval No. K2024-12-089) strictly prior to the commencement of patient enrollment. The trial was conducted in accordance with the Declaration of Helsinki and reported following CONSORT guidelines. All participants provided written informed consent before enrollment.

### Study population

Eligible participants were adults 18 to 80 years of age with stable chronic HF (symptom stability ≥ 3 months). Inclusion criteria required an LVEF < 50% confirmed by echocardiography, encompassing patients with HFrEF (LVEF ≤ 40%) and HFmrEF (LVEF 41–49%). All participants were in New York Heart Association (NYHA) functional class II or III and were physically able to undergo exercise testing and training. Key exclusion criteria were unstable angina or acute myocardial infarction within the preceding 3 months; uncontrolled arrhythmias; hemodynamically significant valvular heart disease requiring intervention; moderate-to-severe chronic obstructive pulmonary disease (FEV₁ <50% of predicted); cognitive impairment; or musculoskeletal conditions precluding participation.

### Randomization and blinding

After baseline assessment, eligible participants were randomly assigned in a 1:1 ratio to the aerobic plus resistance training group (AR group) or the combined ARIS group. The allocation sequence was generated by an independent statistician using a computer-generated random-number sequence. Allocation concealment was ensured using consecutively numbered, opaque, sealed envelopes, which were opened only after a patient was officially enrolled. The study used a PROBE (Prospective Randomized Open Blinded End-point) design. Although participants and supervising therapists could not be blinded to the exercise intervention, all outcome assessors (including echocardiographers and personnel administering the 6MWD and KCCQ) and data analysts were strictly blinded to treatment allocation to eliminate detection bias.

### Interventions

All participants received guideline-directed medical therapy. The exercise program consisted of 36 supervised sessions conducted three times weekly over a nominally prescribed 12-week period.

#### Aerobic and Resistance Training (AR group)

The AR group followed a standardized 60-minute protocol:

(i) Warm-up (5 min): Low-intensity calisthenics.
(ii) Aerobic Training (30–40 min): Cycle ergometry or treadmill walking at 40–59% of heart rate reserve (HRR) [7]. The peak heart rate achieved during the baseline 6MWT under ECG monitoring was utilized as an individualized surrogate for maximal heart rate. The target Borg Rating of Perceived Exertion (RPE) was 12–13. Duration progressed from 30 min at baseline to 40 min by week 6.
(iii) Resistance Training (20–30 min): Exercises targeted major muscle groups (chest press, seated row, biceps/triceps curls, leg press/curl) using dumbbells, resistance bands, and body weight. Patients performed 4 to 6 distinct exercises per session. Following a familiarization session, participants completed 2 to 3 sets of 10 to 15 repetitions, with 1 to 2 min rest intervals. A correct repetition was strictly defined as completing the full range of motion without compensatory momentum. For upper extremity exercises, initial loads were 40–60% of an estimated 1-repetition maximum (1RM) [7, 8], derived from a submaximal test using the Brzycki Equation [18]. For heavy lower-limb exercises, 1RM testing was deferred to avoid the Valsalva maneuver; intensity was instead titrated using the Borg RPE scale (target 11–13).
(iv) Cool-down (5 min): Low-intensity stretching.

Crucially, average exercise duration, training heart rate, lifted weights, and session-RPE were meticulously documented in patient-specific training logs to validate protocol fidelity.

#### ARIS strategy (Combined training)

The ARIS group followed a time-additive strategy. Participants performed the identical AR protocol described above with the addition of a 30-minute respiratory module:

(i) Inspiratory Muscle Training (IMT) (20 min): IMT was performed using a portable resistance device (XEEK, China). Building upon the structural framework established by the ARISTOS-HF trial [16], the 20-minute session was divided into 4 sets of 5 min of continuous loaded breathing, with 1 to 2 min rest intervals. Crucially, rather than initiating training at a high intensity, we employed a progressive loading strategy to optimize patient safety and tolerability. Following established protocols [19], the initial threshold resistance was set at 30% of the patient’s maximal inspiratory pressure (MIP). To maintain training at a precise percentage of the patient’s true capacity, MIP was systematically re-evaluated every 4 weeks. The resistance on the device was manually recalibrated to progressively reach a target intensity of 60% of the newly measured absolute MIP [16].
(ii) Breathing Pattern Retraining (10 min): Included diaphragmatic and pursed-lip breathing.

### Assessment of training adherence and fidelity

Full protocol adherence was strictly defined as the completion of all 36 prescribed sessions. Furthermore, to account for delayed or irregular training patterns commonly observed in real-world clinical settings, we calculated a realistic, time-adjusted training density (actual chronological attendance). As recommended by methodological best practices, this metric (sessions per week) was calculated by dividing the total number of completed sessions by the actual duration in weeks (total days divided by 7) elapsed between the patient’s first session and their final post-intervention assessment.

### Outcome measures

Baseline assessments were performed prior to randomization. Post-intervention outcomes were evaluated at the completion of the 36 prescribed sessions. To accommodate realistic delayed training patterns, assessments occurred at the nominal 12-week mark or within a permitted follow-up window upon session completion.

#### Primary and secondary endpoints

The primary endpoint was the change in KCCQ Overall Summary Score. A change of ≥ 5 points was considered clinically meaningful [20]. Secondary endpoints included: (1) functional capacity assessed by the 6-minute walk distance (6MWD), for which a change of ≥ 30 m was considered clinically meaningful [21]; (2) inspiratory muscle strength (MIP); (3) KCCQ subscores; and (4) cardiac remodeling parameters (LVEF, LVEDV).

#### Echocardiography

Transthoracic echocardiography was performed at baseline and post-intervention by a single dedicated physician who was strictly blinded to group allocations, eliminating inter-rater variability. LVEF and LVEDV were calculated using the modified Simpson’s biplane method from apical 4- and 2-chamber views in strict accordance with ASE guidelines [22].

#### Functional capacity (6MWD)

Assessed in a quiet, straight, 30-meter unimpeded corridor according to ATS guidelines [23]. Standard verbal encouragement was provided every minute by the same evaluator, who remained stationed near the starting line rather than walking behind the patient.

#### Inspiratory muscle strength (MIP

Measured using a digital laboratory spirometer (MasterScreen, Jaeger, Germany) with an integrated P0.1 testing software module for standardized data acquisition. Subjects exhaled to residual volume (RV), followed by a maximal explosive inspiratory effort (Müller maneuver) against an occluded airway for at least 1.5 s. The highest of three reproducible readings (< 10% variability) was recorded.

### Sample size determination

The sample size was formally calculated based on the expected effect size derived from the reference ARISTOS-HF trial [16], which demonstrated a mean difference of 7.79 to 8.96 points in quality of life. Hypothesizing a conservative between-group superiority of 7.5 points for the KCCQ score (SD = 10.0), a minimum of 28 patients per group was required (80% power, α = 0.05). Allowing for a 15% dropout rate, the target was 33 patients per group (total *n* = 66).

### Statistical analysis

Analyses were performed strictly following the intention-to-treat (ITT) principle, guided by established biostatistical frameworks for clinical research [24]. For the primary outcome (KCCQ score), 100% follow-up was achieved; data for the 5 patients unable to attend the final clinical visit were successfully obtained via structured telephone interviews. For secondary clinic-based outcomes (e.g., 6MWD, MIP, and echocardiography), 5 missing values (AR: *n* = 3; ARIS: *n* = 2) were handled using Multiple Imputation by Chained Equations (MICE, 5 imputations) under the missing-at-random assumption.

Continuous variables were assessed for normality using the Shapiro-Wilk test. Because the changes from baseline (Deltas) exhibited non-normal distributions, between-group comparisons were robustly conducted using the non-parametric Mann-Whitney U test. For MICE-analyzed outcomes, the most conservative P-value across the 5 imputations is reported. To facilitate clinical interpretability, between-group mean differences and 95% confidence intervals (CIs) were derived from parametric estimations.

For within-group comparisons (baseline vs. post-intervention), paired t-tests were utilized. For variables subjected to MICE, the within-group paired t-test results were pooled across the 5 imputed datasets according to Rubin’s rules. A two-sided *P*-value < 0.05 was considered statistically significant. Statistical analyses were performed using SPSS software.

## Results

### Study population and adherence

**Fig. 1:**
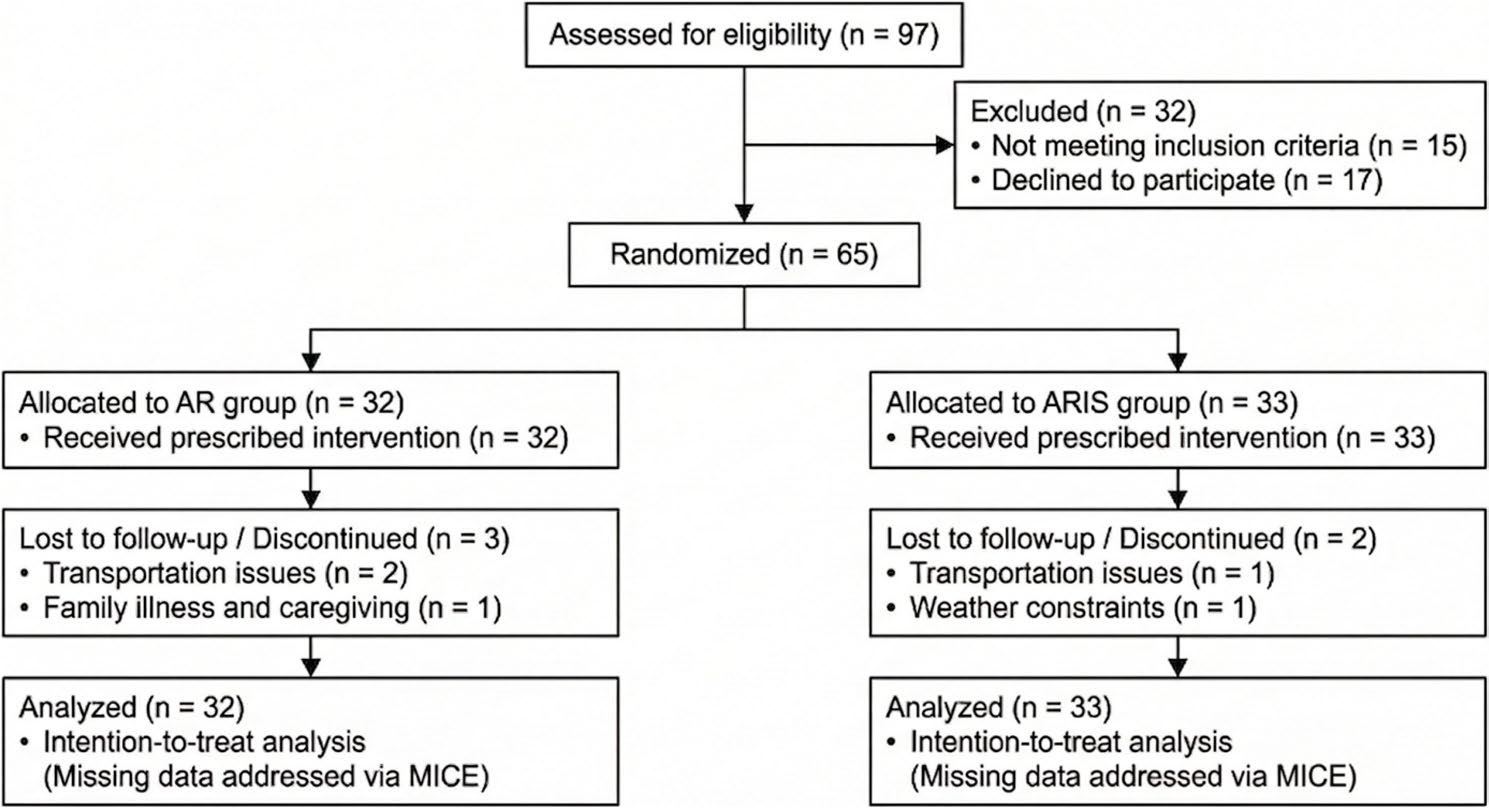
CONSORT flow diagram of the study

A total of 65 patients were enrolled and randomly assigned to either the standard aerobic and resistance training group (AR group, *n* = 32) or the combined training group incorporating inspiratory muscle training (ARIS group, *n* = 33) (Fig. 1). Baseline demographic and clinical characteristics, including cardiac function and medical therapy, were well balanced between the two cohorts (Table 1).

**Table 1:** Baseline characteristics of the study population

Characteristics	AR Group (*n* = 32)	ARIS Group (*n* = 33)	*P* Value
Demographics			
Age — yr	56.3 ± 16.0	53.7 ± 14.1	0.49
Male sex — no. (%)	26 (81.3)	27 (81.8)	0.95
Body-mass index — kg/m²	23.1 ± 4.1	23.3 ± 4.3	0.85
Clinical Characteristics			
Resting Heart rate — beats/min	78.1 ± 13.5	79.0 ± 14.9	0.80
Resting Systolic blood pressure — mm Hg	116.1 ± 17.4	115.3 ± 14.2	0.84
Resting Diastolic blood pressure — mm Hg	68.8 ± 9.1	72.9 ± 11.3	0.12
NYHA functional class — no. (%)			0.66
Class II	10 (31.3)	12 (36.4)	
Class III	22 (68.8)	21 (63.6)	
Comorbidities — no. (%)			
Hypertension	14 (43.8)	14 (42.4)	0.91
Diabetes mellitus	6 (18.8)	7 (21.2)	0.80
Hyperlipidemia	7 (21.9)	9 (27.3)	0.61
Current or former smoker	18 (56.3)	20 (60.6)	0.72
Etiology of Heart Failure — no. (%)			
Coronary artery disease	8 (25.0)	13 (39.4)	0.22
Cardiomyopathy	14 (43.8)	20 (60.6)	0.17
Valvular heart disease	14 (43.8)	14 (42.4)	0.91
Atrial fibrillation	11 (34.4)	8 (24.2)	0.37
Medications — no. (%)			
Beta-blockers	24(75.0)	29 (87.9)	0.22
RAS inhibitors	26 (81.3)	24 (72.7)	0.17
Mineralocorticoid receptor antagonists	28 (87.5)	25 (75.8)	0.22
SGLT2 inhibitors	21 (65.6)	18 (54.5)	0.36
Diuretics	27 (84.4)	27 (81.8)	0.78
Vericiguat	6 (18.8)	8 (24.2)	0.59
Echocardiographic Parameters			
LVEF — %	32.4 ± 7.7	34.2 ± 9.0	0.37
HFmrEF no. (%)	6(18.8)	8(24.2)	0.76
LVEDV — mL	173.0 ± 50.2	179.9 ± 74.7	0.66
LV end-diastolic diameter — mm	62.1 ± 9.5	62.0 ± 10.0	0.989
Functional Capacity and Quality of Life			
MIP (kPa)	5.7 ± 2.3	5.5 ± 1.7	0.796
6-Minute walk distance — m	396.1 ± 76.5	390.6 ± 86.2	0.79
KCCQ Scores			
Overall Summary Score	58.5 ± 14.5	57.6 ± 14.5	0.80
Clinical Summary Score	63.3 ± 15.6	64.9 ± 15.0	0.66
Symptom Burden	65.0 ± 17.4	64.4 ± 16.1	0.89
Physical Limitation	61.5 ± 16.6	65.4 ± 17.6	0.36
Self-Efficacy	53.6 ± 16.7	54.6 ± 19.5	0.83
Social Limitation	58.4 ± 21.6	55.7 ± 18.7	0.59
Quality of Life	49.2 ± 20.8	44.9 ± 18.1	0.38

Plus-minus values are means ± SD. Percentages may not total 100 because of rounding. RAS inhibitors included angiotensin-converting-enzyme (ACE) inhibitors, angiotensin-receptor blockers (ARBs), and angiotensin receptor–neprilysin inhibitors (ARNIs). KCCQ scores ranged from 0 to 100, with higher scores indicating better health status. A change of 5 points was considered clinically significant

*AR* denotes aerobic and resistance training, *ARIS* Aerobic resistance and IMT, *LVEF* Left ventricular ejection fraction, *LVEDV* Left ventricular end-diastolic volume, *NYHA* New York Heart Association, *SGLT2* Sodium–glucose cotransporter 2, *KCCQ* Kansas City Cardiomyopathy Questionnaire

A total of 97 patients were assessed for eligibility, of whom 65 were randomized to either the standard aerobic and resistance training (AR) group (*n* = 32) or the combined ARIS strategy group (*n* = 33). All randomized patients were included in the final intention-to-treat (ITT) analysis. Missing outcome data due to study discontinuation or loss to follow-up were strictly handled using Multiple Imputation by Chained Equations (MICE) to prevent attrition bias.

Regarding intervention adherence, the mean number of completed training sessions was similar between the groups (32.6 ± 5.5 in the AR group vs. 32.8 ± 5.5 in the ARIS group; *P* = 0.85). Although full protocol adherence—strictly defined as completing all 36 prescribed sessions—was achieved by only 53.1% of patients in the AR group and 57.6% in the ARIS group (*P* = 0.72), a more rigorous, time-adjusted fidelity metric was applied to account for irregular or delayed training patterns. This analysis demonstrated a realistic chronological attendance of 2.38 ± 0.64 sessions per week in the AR group and 2.53 ± 0.70 sessions per week in the ARIS group (*P* = 0.370). This equated to an actual adherence rate of approximately 79.3% and 84.3%, respectively, relative to the targeted 3 sessions per week. Following the intention-to-treat (ITT) principle, all 65 randomized patients were included in the primary outcome analysis.

### Primary outcome: quality of life

At the post-intervention evaluation, both groups demonstrated marked within-group improvements in the primary outcome compared to baseline. Crucially, the between-group analysis of mean changes revealed that the addition of IMT in the ARIS group yielded a significantly greater improvement in the KCCQ Overall Summary Score than standard rehabilitation alone (mean change: 19.8 ± 10.5 vs. 13.5 ± 8.2; Mean Difference [MD]: 6.3, 95% CI: 1.6 to 10.9; *P* = 0.006). Notably, this between-group mean difference (MD: 6.3) exceeded the established minimal clinically important difference (MCID) of 5 points.

### Secondary outcomes: functional capacity and domain-specific analysis

Based on the ITT analysis utilizing multiple imputation (MICE) for missing data, the ARIS intervention led to significant between-group superiority in both functional capacity and respiratory muscle strength. The ARIS group achieved a substantially greater increase in the 6-Minute Walk Distance compared to the AR group (mean change: 89.0 ± 56.4 m vs. 55.1 ± 54.4 m; MD: 33.9 m, 95% CI: 6.4 to 61.4; *P* = 0.016) (Table 2). Notably, this inter-group mean difference also exceeded the widely recognized clinically meaningful threshold of 30 m. Concurrently, maximal inspiratory pressure (MIP) was remarkably enhanced in the ARIS group (mean change: 3.2 ± 1.4 kPa vs. 1.2 ± 1.0 kPa; MD: 2.0 kPa, 95% CI: 1.4 to 2.6; *P* < 0.001).

**Table 2:** Primary and secondary outcomes from baseline to post-intervention

Outcome Measures	AR Group (*n* = 32)			ARIS Group (*n* = 33)			Between-Group Difference	
	Baseline	Post-intervention	Mean Change	Baseline	Post-intervention	Mean Change	Mean Diff. (95% CI)	*P* Value
Primary Outcome								
KCCQ Overall Summary Score	58.5 ± 14.5	72.0 ± 10.1*	13.5 ± 8.2	57.6 ± 14.5	77.4 ± 8.9*	19.8 ± 10.5	6.3 (1.6 to 10.9)	**0.006**
Secondary Outcomes								
6-Minute Walk Dist. (m)	396.1 ± 76.5	451.2 ± 75.2*	55.1 ± 54.4	390.6 ± 86.2	479.6 ± 64.8*	89.0 ± 56.4	33.9 (6.4 to 61.4)	**0.016**
MIP (kPa)	5.7 ± 2.3	6.9 ± 2.4*	1.2 ± 1.0	5.5 ± 1.7	8.7 ± 2.0*	3.2 ± 1.4	2.0 (1.4 to 2.6)	**< 0.001**
KCCQ Domains								
Clinical Summary Score	63.3 ± 15.6	74.3 ± 11.4*	11.0 ± 8.5	64.9 ± 15.0	80.8 ± 10.9*	15.9 ± 11.9	4.9 (-0.3 to 10.1)	0.136
Symptom Burden	65.0 ± 17.4	74.5 ± 11.4*	9.5 ± 10.1	64.4 ± 16.1	80.1 ± 10.9*	15.7 ± 14.2	6.3 (0.1 to 12.4)	0.126
Physical Limitation	61.5 ± 16.6	74.0 ± 14.3*	12.5 ± 9.2	65.4 ± 17.6	81.4 ± 13.8*	16.0 ± 13.4	3.6 (-2.2 to 9.3)	0.168
Self-Efficacy	53.6 ± 16.7	66.8 ± 15.4*	13.2 ± 6.9	54.6 ± 19.5	77.7 ± 12.8*	23.1 ± 18.3	9.9 (2.9 to 16.9)	**0.012**
Social Limitation	58.4 ± 21.6	74.0 ± 15.7*	15.6 ± 12.0	55.7 ± 18.7	74.6 ± 12.4*	18.9 ± 13.4	3.2 (-3.1 to 9.5)	0.505
Quality of Life	49.2 ± 20.8	65.8 ± 16.3*	16.6 ± 11.3	44.9 ± 18.1	73.5 ± 12.6*	28.6 ± 14.5	12.0 (5.5 to 18.5)	**0.001**
Echocardiography								
LVEF (%)	32.4 ± 7.7	38.8 ± 11.2*	6.4 ± 8.1	34.2 ± 9.0	41.1 ± 13.2*	6.9 ± 9.5	0.5 (-3.9 to 4.8)	0.818
LVEDV (mL)	173.0 ± 50.2	168.2 ± 65.2	-4.8 ± 65.7	179.9 ± 74.7	164.4 ± 78.8*	-15.5 ± 40.4	-10.8 (-37.7 to 16.2)	0.990
LVEDd (mm)	62.1 ± 9.5	59.7 ± 9.1*	-2.3 ± 6.2	62.0 ± 10.0	59.7 ± 10.7*	-2.3 ± 6.5	0 (-3.2 to 3.2)	0.953

Data are presented as mean ± standard deviation (SD). Bold font indicates statistical significance (*P*< 0.05). Missing values at post-intervention were handled via Multiple Imputation by Chained Equations (MICE, 5 imputations). *P*-values for between-group differences were universally calculated using the non-parametric Mann-Whitney U test on the change scores (deltas). For variables requiring MICE, the most conservative *P*-value across the 5 imputations is reported. To facilitate clinical interpretability, the between-group mean differences and 95% confidence intervals (CIs) are derived from parametric estimations. Minor discrepancies between the reported mean differences and the calculated differences from the displayed means are due to rounding

*P < 0.05 for within-group comparisons (post-intervention vs. baseline), evaluated using paired t-tests (for MICE data, results were pooled across the 5 imputed datasets using Rubin’s rules)

**Fig. 2:**
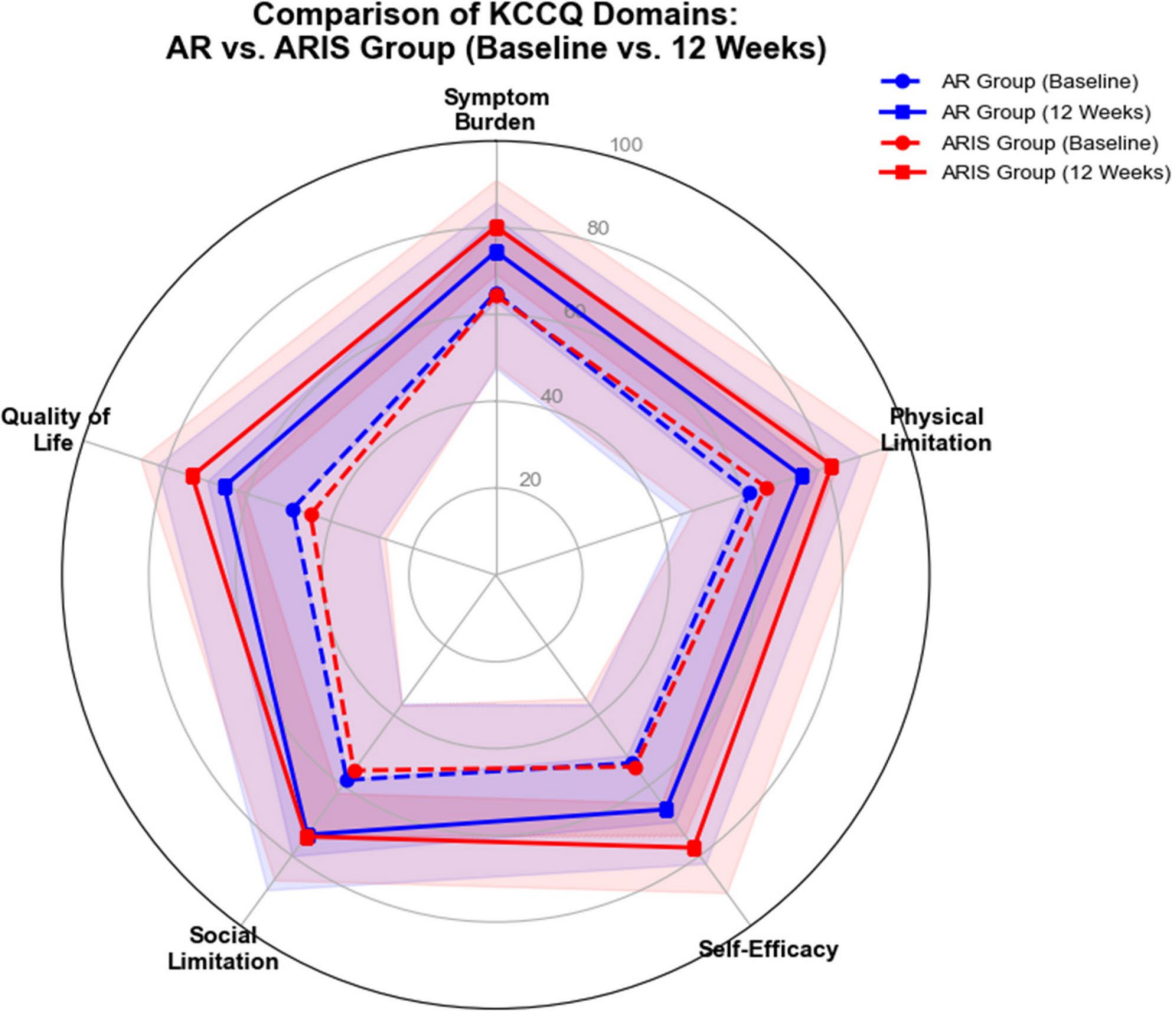
Radar chart illustrating the multidimensional impact of rehabilitation on KCCQ domains

Further domain-specific KCCQ analysis elucidated the multidimensional benefits of the intervention (Fig. 2). The ARIS group demonstrated statistically significant superiority over the AR group in the mean changes for Self-Efficacy (mean change: 23.1 ± 18.3 vs. 13.2 ± 6.9; MD: 9.9, 95% CI: 2.9 to 16.9; *P* = 0.012) and Quality of Life (mean change: 28.6 ± 14.5 vs. 16.6 ± 11.3; MD: 12.0, 95% CI: 5.5 to 18.5; *P* = 0.001). Although numerical trends favoring the ARIS group were also observed in the Clinical Summary Score, Symptom Burden, Physical Limitation, and Social Limitation domains, these differences did not reach statistical significance under the conservative non-parametric assessments (all *P* > 0.05).

The plot shows the mean scores for the five KCCQ domains at baseline (dashed lines) and at 12 weeks (solid lines). Blue lines represent the AR group, and red lines represent the ARIS group. Shaded areas indicate 95% confidence intervals. A more pronounced expansion was observed in the Quality of Life, Self-Efficacy, and Symptom domains in the ARIS group than in the AR group, illustrating the domain-specific benefits of respiratory muscle training.

### Cardiac structure and function

By the end of the rehabilitation program, significant within-group improvements were observed in specific structural and functional echocardiographic parameters. However, between-group comparisons based on the pooled imputed data indicated that the magnitude of these changes was statistically comparable between the ARIS and AR groups, with no significant differences observed in the mean changes for LVEF (*P* = 0.818), LVEDV (*P* = 0.990), or LVEDd (*P* = 0.953) (Table 2).

To further investigate potential additive structural benefits in patients with more advanced dysfunction, a prespecified subgroup analysis was conducted in patients with a baseline LVEF < 35% (total *n* = 38). Consistent with the primary methodology, missing data at post-intervention (*n* = 3) were handled using MICE. In this severely impaired subgroup, both the AR and ARIS groups achieved significant within-group improvements in LVEF. However, regarding ventricular geometry, a significant within-group reduction in LVEDd was observed only in the standard AR group, whereas the change in the ARIS group was not statistically significant. Overall, the between-group comparisons of mean changes within this subgroup revealed no statistically significant differences in LVEF (*P* = 0.593), LVEDV (*P* = 0.874), or LVEDd (*P* = 0.763) (Supplementary Table S1).

### Safety

The safety profile was highly favorable in both training arms. No deaths, serious adverse events, or unplanned hospitalizations for worsening heart failure occurred during the study period. All reported adverse events were minor (e.g., transient muscle soreness) and resolved spontaneously without necessitating interruptions to the training protocols.

## Discussion

### Principal findings

This randomized controlled trial demonstrated that in patients with HF across a broad spectrum of reduced ejection fraction (LVEF < 50%), the addition of IMT to a full-dose standard aerobic and resistance (AR) protocol yielded clinically meaningful incremental benefits. The principal finding was that the time-additive ARIS strategy resulted in a significantly greater improvement in the KCCQ Overall Summary Score (between-group difference: +6.3 points) and the 6MWD (+ 33.9 m) compared to standard training alone. The magnitude of these benefits exceeded established thresholds for clinical significance (5 points for KCCQ and 30 m for 6MWD). Importantly, achieving a > 30-meter increase in the 6MWD represents a robust minimal clinically important difference (MCID) that has been independently associated with significant reductions in long-term mortality and heart failure-related rehospitalizations in previous literature [25, 26].

These findings indicate that targeting impaired inspiratory muscle strength provides robust additional therapeutic benefits over conventional rehabilitation models. Notably, while statistically significant between-group differences in reverse remodeling parameters (LVEF or LVEDV) were not observed, the significant within-group improvements—particularly the more pronounced reduction in LVEDV in the ARIS group—suggest that these profound clinical gains are likely driven by a synergistic combination of peripheral respiratory adaptations and early signs of structural cardiac recovery.

The improvement in functional capacity and quality of life is mechanistically supported by enhanced respiratory muscle strength. A substantial increase in maximal inspiratory pressure (MIP) was observed in the ARIS group compared to the AR group (between-group difference: 2.0 kPa). This finding confirms that the time-additive IMT protocol effectively reversed impaired inspiratory muscle strength—a common comorbidity and functional limiter in this population.

### Comparison with previous studies and methodological advances

The landmark ARISTOS-HF trial successfully implemented combined rehabilitation training utilizing a rigorous isochronal (time-matched) design. It convincingly demonstrated that the ARIS strategy still yielded greater rehabilitation benefits for patients with heart failure, even when the duration of standard training was proportionally shortened to accommodate IMT [16]. By employing a novel “time-additive” strategy, our study extends the ARISTOS-HF trial to address a critical question: how much incremental benefit can be achieved by superimposing IMT and extending the total rehabilitation duration on top of a full-dose standard rehabilitation protocol?

Answering this question, the ARIS group in our study achieved a profound within-group improvement of 89.0 m in the 6MWD. Compared to the robust 72.2-meter gain reported in the ARISTOS-HF trial, achieving a relatively larger magnitude reinforces the core premise highlighted by ARISTOS-HF—that exercise time and volume themselves are important drivers of patient improvement [16]. While this 89.0-meter gain exceeds the typical 30–50 m often reported in traditional, single-modality heart failure rehabilitation literature, it aligns closely with the results of other multi-domain intervention trials. For instance, the REHAB-HF trial reported a remarkably similar within-group increase of 99 m in the 6MWD [15].

### Mechanisms of additive benefit: a synergistic perspective

The marked clinical gains in the ARIS group are likely attributable to the combined effects of a higher overall exercise volume and the complementary benefits of targeted IMT. In patients with HF, impaired inspiratory muscles are prone to early fatigue during exertion, which triggers a sympathetically mediated respiratory metaboreflex. This reflex induces peripheral vasoconstriction, prematurely restricting blood flow to locomotor muscles [10, 12, 14]. By effectively enhancing inspiratory muscle strength, the IMT intervention may attenuate this metaboreflex. Consequently, delaying respiratory fatigue helps preserve limb perfusion, which directly translates into extended walking distance and delayed exertional fatigue.

Beyond physiological hemodynamics, our domain-specific KCCQ analysis revealed that the significant between-group superiority was primarily observed in the Quality of Life and Self-Efficacy domains, rather than the symptom frequency domain. This highlights a critical psychological mechanism. While overall symptom relief may be comparable between groups, the targeted enhancement of respiratory muscle strength, coupled with the generalized physical conditioning from an extended exercise duration, synergistically boosts patient confidence. This psychological empowerment helps disrupt the debilitating “dyspnea–anxiety” cycle [27, 28].

### Cardiac reverse remodeling and exercise volume

After receiving optimized guideline-directed medical therapy (GDMT), patients undergoing both training modalities achieved significant within-group improvements in left ventricular ejection fraction (LVEF). The active role of exercise rehabilitation must be considered when interpreting these positive structural adaptations. As discussed by the investigators of the ARISTOS-HF trial [16], achieving a robust weekly training duration (e.g., 180 min/week) appears to be a critical parameter for facilitating cardiac improvements.

However, our rigorous statistical analysis—incorporating multiple imputation for missing data—revealed no significant between-group differences in the mean changes for LVEF, LVEDV, or LVEDd. While a numerically larger reduction in LVEDV was observed within the ARIS group (mean change: -15.5 mL vs. -4.8 mL in the AR group), the lack of statistical significance in between-group comparisons indicates that the addition of IMT did not yield a statistically robust additive benefit for cardiac reverse remodeling. Furthermore, our prespecified subgroup analysis confirmed that even in patients with more severe baseline dysfunction (LVEF < 35%), the extended ARIS strategy did not translate into superior structural recovery compared to standard training (Supplementary Table S1). This may be partially attributable to the insufficient follow-up duration. The 12-week intervention period is relatively short to induce massive between-group structural remodeling, which typically requires prolonged hemodynamic unloading (> 6 months) [29]. Therefore, achieving an extended exercise duration may be a critical parameter for maximizing cardiac structural improvement, and further studies with extended follow-up are warranted to definitively evaluate the long-term impact of the ARIS strategy on cardiac geometry.

### Training adherence and patient preference

Finally, the relatively low overall adherence rate (< 60%) in our trial warrants discussion. This highlights the inherent real-world challenge for HF patients to strictly maintain a facility-based, 3-day-per-week training schedule over a 12-week period. Interestingly, adherence was numerically superior in the ARIS group. However, when applying a realistic, time-adjusted fidelity metric, the actual chronological attendance was robust (averaging 2.38 and 2.53 sessions per week in the AR and ARIS groups, respectively). Interestingly, adherence was numerically superior in the ARIS group. This phenomenon is consistent with the programme preference survey (PPS) results reported in the ARISTOS-HF trial [16], which indicated that patients found multimodality training (combining aerobic, resistance, and IMT) significantly more engaging and less monotonous than conventional single-modality continuous exercise. To translate these multimodality benefits to broader clinical practice and overcome attendance barriers, future strategies should strongly consider integrating hybrid home-based models or tele-rehabilitation with remote monitoring.

### Limitations

This study has several limitations. First and foremost, the major limitation is the difference in total exercise time between the groups, an inherent consequence of our time-additive design. Future studies should incorporate a sham-IMT protocol (e.g., at 10% MIP) in the standard AR group to ensure equal exercise duration and isolate the specific effects of inspiratory resistance. Second, it was conducted at a single center with a relatively small sample size, limiting generalizability. Third, the lack of cardiopulmonary exercise testing (CPET) prevented us from assessing peak oxygen consumption (VO2peak), a gold-standard prognostic marker.

## Conclusions

In patients with HF (LVEF < 50%), the time-additive integration of IMT into a standard aerobic and resistance training protocol yielded clinically meaningful incremental benefits in functional capacity and health-related quality of life. While the synergistic strategy significantly improved walking distance and self-efficacy, it did not provide statistically significant additional benefits for cardiac reverse remodeling compared to high-volume standard training alone. Multimodality training appears more engaging, but broader implementation will require innovative delivery models to optimize long-term adherence.

## Supplementary Information

Supplementary Material 1

## Data Availability

The datasets used and/or analyzed during the current study are available from the corresponding author on reasonable request.
