# Supplementary material for: Inspiratory muscle training as an adjunct to standard cardiac rehabilitation in patients with LVEF < 50%: a randomized controlled trial
Source: BMC Cardiovasc Disord. 2026 Apr 21;26:474. doi: 10.1186/s12872-026-05889-4 (PMC13231551; doi:10.1186/s12872-026-05889-4)
Supplement: Supplementary file 1 — Supplementary Material 1 [file 12872_2026_5889_MOESM1_ESM.pdf]

CONSORT 2010 checklist of information to include when reporting a randomised trial*

Section/Topic	Item No	Checklist item	Reported on page No
**Title and abstract**			
	1a	Identification as a randomised trial in the title	NO1
	1b	Structured summary of trial design, methods, results, and conclusions (for specific guidance see CONSORT for abstracts)	NO1
**Introduction**			
Background and	2a	Scientific background and explanation of rationale	NO 2
objectives	2b	Specific objectives or hypotheses	N0 2
**Methods**			
Trial design	3a	Description of trial design (such as parallel, factorial) including allocation ratio	NO 3
	3b	Important changes to methods after trial commencement (such as eligibility criteria), with reasons	Not applicable
Participants	4a	Eligibility criteria for participants	NO3
	4b	Settings and locations where the data were collected	NO3
Interventions	5	The interventions for each group with sufficient details to allow replication, including how and when they were actually administered	NO3-4
Outcomes	6a	Completely defined pre-specified primary and secondary outcome measures, including how and when they were assessed	NO4
	6b	Any changes to trial outcomes after the trial commenced, with reasons	Not applicable
Sample size	7a	How sample size was determined	NO4
	7b	When applicable, explanation of any interim analyses and stopping guidelines	Not applicable
Randomisation:			
Sequence generation	8a	Method used to generate the random allocation sequence	NO3
	8b	Type of randomisation; details of any restriction (such as blocking and block size)	NO3
Allocation concealment mechanism	9	Mechanism used to implement the random allocation sequence (such as sequentially numbered containers), describing any steps taken to conceal the sequence until interventions were assigned	NO3
Implementation	10	Who generated the random allocation sequence, who enrolled participants, and who assigned participants to interventions	NO3
Blinding	11a	If done, who was blinded after assignment to interventions (for example, participants, care providers, those	NO3

		assessing outcomes) and how	
	11b	If relevant, description of the similarity of interventions	Not applicable
Statistical methods	12a	Statistical methods used to compare groups for primary and secondary outcomes	NO4
	12b	Methods for additional analyses, such as subgroup analyses and adjusted analyses	NO4
**Results**			
Participant flow (a diagram is strongly recommended)	13a	For each group, the numbers of participants who were randomly assigned, received intended treatment, and were analysed for the primary outcome	NO4
	13b	For each group, losses and exclusions after randomisation, together with reasons	NO4-5
Recruitment	14a	Dates defining the periods of recruitment and follow-up	No5
	14b	Why the trial ended or was stopped	NO3
Baseline data	15	A table showing baseline demographic and clinical characteristics for each group	NO5-7
Numbers analysed	16	For each group, number of participants (denominator) included in each analysis and whether the analysis was by original assigned groups	NO7
Outcomes and estimation	17a	For each primary and secondary outcome, results for each group, and the estimated effect size and its precision (such as 95% confidence interval)	NO7-8
	17b	For binary outcomes, presentation of both absolute and relative effect sizes is recommended	Not applicable
Ancillary analyses	18	Results of any other analyses performed, including subgroup analyses and adjusted analyses, distinguishing pre-specified from exploratory	NO7-8
Harms	19	All important harms or unintended effects in each group (for specific guidance see CONSORT for harms)	NO5
**Discussion**			
Limitations	20	Trial limitations, addressing sources of potential bias, imprecision, and, if relevant, multiplicity of analyses	NO10
Generalisability	21	Generalisability (external validity, applicability) of the trial findings	NO10
Interpretation	22	Interpretation consistent with results, balancing benefits and harms, and considering other relevant evidence	NO8-10
**Other information**			
Registration	23	Registration number and name of trial registry	NO3
Protocol	24	Where the full trial protocol can be accessed, if available	Not applicable
Funding	25	Sources of funding and other support (such as supply of drugs), role of funders	NO11

*We strongly recommend reading this statement in conjunction with the CONSORT 2010 Explanation and Elaboration for important clarifications on all the items. If relevant, we also recommend reading CONSORT extensions for cluster randomised trials, non-inferiority and equivalence trials, non-pharmacological treatments, herbal interventions, and pragmatic trials. Additional extensions are forthcoming: for those and for up to date references relevant to this checklist, see www.consort-statement.org.
